# Association between spirometry controlled chest CT scores using computer-animated biofeedback and clinical markers of lung disease in children with cystic fibrosis

**DOI:** 10.1080/20018525.2017.1318027

**Published:** 2017-05-02

**Authors:** Thomas Kongstad, Kent Green, Frederik Buchvald, Marianne Skov, Tania Pressler, Kim Gjerum Nielsen

**Affiliations:** ^a^ CF Center Copenhagen, Paediatric Pulmonary Service, Department of Paediatrics and Adolescent Medicine, Copenhagen University Hospital, Rigshospitalet, Copenhagen, Denmark; ^b^ Research Unit on Women’s and Children’s Health, Copenhagen University Hospital, Rigshospitalet, Copenhagen, Denmark

**Keywords:** Spirometry controlled chest CT, cystic fibrosis, *Aspergillus*, LCI

## Abstract

**Background**: Computed tomography (CT) of the lungs is the gold standard for assessing the extent of structural changes in the lungs. Spirometry-controlled chest CT (SCCCT) has improved the usefulness of CT by standardising inspiratory and expiratory lung volumes during imaging. This was a single-centre cross-sectional study in children with cystic fibrosis (CF). Using SCCCT we wished to investigate the association between the quantity and extent of structural lung changes and pulmonary function outcomes, and prevalence of known CF lung pathogens.

**Methods**: CT images were analysed by CF-CT scoring (expressed as % of maximum score) to quantify different aspects of structural lung changes including bronchiectasis, airway wall thickening, mucus plugging, opacities, cysts, bullae and gas trapping. Clinical markers consisted of outcomes from pulmonary function tests, microbiological cultures from sputum and serological samples reflecting anti-bacterial and anti-fungal antibodies.

**Results**: Sixty-four children with CF, median age (range) of 12.7 (6.4–18.1) years, participated in the study. The median (range) CF-CT total score in all children was 9.3% (0.4–46.8) with gas trapping of 40.7% (3.7–100) as the most abundant finding. Significantly higher median CF-CT total scores (21.9%) were found in patients with chronic infections (*N *= 12) including Gram-negative infection and allergic bronchopulmonary aspergillosis (ABPA) exhibiting CF-CT total scores of 14.2% (ns) and 24.0% (*p* < 0.01), respectively, compared to 8.0% in patients with no chronic lung infection. Lung clearance index (LCI) derived from multiple breath washout exhibited closest association with total CF-CT scores, compared to other pulmonary function outcomes.

**Conclusions**: The most prominent structural lung change was gas trapping, while CF-CT total scores were generally low, both showing close association with LCI. Chronic lung infections, specifically in the form of ABPA, were associated with increased scores in lung changes. Further investigation of impact of infections with different microorganisms on extent and progression of structural CF lung disease is needed.

## Introduction

Structural lung changes such as bronchiectasis, airway wall thickening, mucus plugging, opacities, cysts, bullae and gas trapping are very common in patients with cystic fibrosis (CF) and caused by a vicious cycle of mucus stagnation, inflammation, and infection.[1,2] Progression of structural lung changes is a sign of worsening lung disease that will eventually lead to increased morbidity and mortality.[3] Timely detection should lead to changes in therapy to stop further progression, and is thus essential to improve the long term outcome.

Computed tomography (CT) of the lungs is the gold standard for assessing the extent of structural changes in the lungs,[4,5] and manoeuvres for controlling breath holds during imaging has further improved the usefulness of CT by standardising lung volumes. This standardisation improves the cross-sectional and longitudinal comparability between groups and within subjects with repeated examinations,[6–8] and increases the recognition of gas trapping.[9]

Monitoring and treating the microbiological pathogens in CF patients is a fundamental part of CF management.[10] The impact of chronic Gram-negative infections on the progression of structural CF lung disease and deteriorating pulmonary function and quality of life is well established.[11–15] Another well-known risk factor for progression of CF lung disease is development of allergic bronchopulmonary aspergillosis (ABPA) [16] whereas the general impact including structural changes of infection with *Aspergillus fumigatus*, allergy or not, is less well substantiated.[17–19]

Spirometry may be a suboptimal tool for monitoring early CF lung disease,[20,21] whereas multiple breath gas washout (MBW) assessing lung clearance index (LCI) seems to be more promising,[22] and may have better sensitivity to detect small airways disease relative to spirometry. The importance of this remains to be validated. However, normal LCI values have been reported in individual CF patients with structural lung changes on CT and vice versa.[23] Therefore, applicability of MBW as complimentary to CT in describing the state of lung disease still needs to be investigated.

The purpose of this study was to investigate the association between the quantity and extent of structural lung changes using refined spirometry controlled chest computed tomography (SCCCT) with computer-animated feedback and clinical markers of CF lung disease such as pulmonary function outcomes, and prevalence of known CF lung pathogens in a cohort of clinically stable CF children.

We hypothesized that children with chronic Gram-negative lung infection or ABPA would present significantly more abundant structural changes. Second, we hypothesized that LCI would serve as the marker with closest association with CF-CT scores, compared to other pulmonary function outcomes.

This study was presented in abstract form as part of workshop 20 (Indexing the lung) at the European Cystic Fibrosis Conference in Gothenburg, 11–14 June 2014.

## Materials and methods

### Study design and inclusion criteria

This study was a cross-sectional, single-occasion, and single-centre study of 6–18 year old children diagnosed with CF according to characteristic clinical features, positive sweat test, and CFTR mutations.[24] All were followed at CF Centre Copenhagen at Rigshospitalet, Copenhagen, Denmark between 2011 and 2012. All clinically stable children, i.e. without signs of exacerbation as defined below, were eligible and consent was obtained from the patient and/or parents. Inclusion was postponed at least 4 weeks after the resolution of symptoms and two or more signs of exacerbation: cough, malaise, dyspnoea, increased sputum production, anorexia, or decline in lung function >10% from baseline values.[25]

In order to evaluate whether the study cohort was representative of the total cohort of children at the centre, a comparison was done between the included and non-included patients according to: (1) lung function (FEV_1_ and FVC); (2) frequency of chronic Gram-negative infections; and (3) level of specific *Pseudomonas aeruginosa* IgG antibodies [26] and markers of *Aspergillus* lung infection (levels of specific anti-*Aspergillus* IgG antibodies) [27] from the same time span as the examinations performed in the study cohort.

### Examinations

The study was performed on a single occasion where patients were examined using SCCCT, pulmonary function tests, and a clinical evaluation by a specialised CF physician.

#### Computed tomography

SCCCT examinations of the lungs were conducted using real-time spirometric monitoring and biofeedback computer software in which inspiratory and expiratory image sequences were captured at lung volumes close to total lung capacity and close to expiratory residual volume, respectively. During CT scanning, lung volumes were displayed in real time on a small monitor simultaneously showing an incentive animation and depicting the threshold volumes of expiratory reserve volume (ERV) and total lung capacity (TLC) as horizontal lines to be reached during successful breath hold, as described previously.[9] Only when required inspiratory and expiratory threshold volumes were reached, image acquisition was initiated by signalling to the CT technician. All scans were performed by volumetric spiral CT imaging on a Toshiba Aquillion 64 CT scanner (Toshiba Corporation, Tokyo, Japan) with 100 kVp, mAs-modulation (SD = 19 in inspiratory sequences and SD = 27 in expiratory sequences, rotation 0.4 s). Additional information and specifications can be found in the Appendix.

#### CF-CT scoring

**Figure A1.**
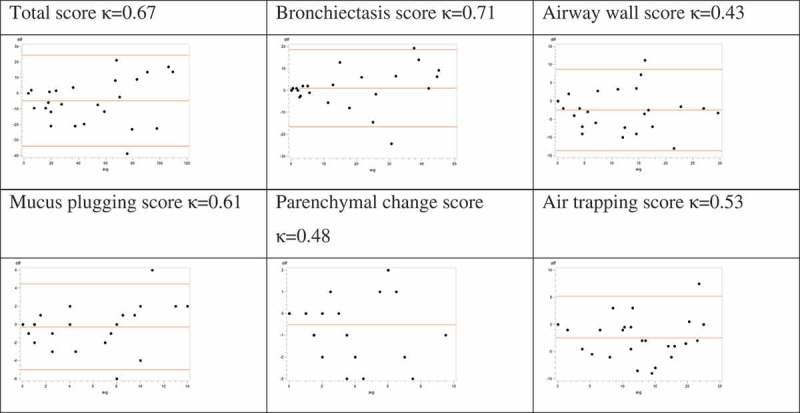
Bland–Altman plots for total score and subdomains assessing systematic bias between scorers in the training CTs done prior to scoring the study cohort.

Structural lung changes visualised on CT were quantified using the CF-CT scoring system for evaluating the extent and severity of central and peripheral bronchiectasis, airway wall thickening, central and peripheral mucus plugging, opacities, cysts and bullae on inspiratory images, and the extent and pattern of gas trapping on expiratory images.[28] Scores were reported as a percentage of the maximum theoretical score of 243 points. Prior to the study, the observer (TK) was trained and certified in the CF-CT scoring system;[29] see Appendix (Figure A1) for details. When scoring the study cohort, the observer was blinded to the clinical backgrounds of the subjects by assigning random unique case numbers to CT examinations prior to scoring.

#### Microbiology and serum markers of infection

Routine clinical data have been collected prospectively in the central database for several years. For this study clinical data from the year prior to the study visit were retrieved from the database. Three groups were identified depending on the infection status: (1) chronic Gram-negative infection with *P. aeruginosa, Achromobacter xylosoxidans* or *Burkholderia cepacia*; (2) ABPA; or (3) Neither 1 nor 2.

Chronic infection was defined and based on growth of the respective bacteria in >50% of months in the previous year and increase in specific IgG antibodies against Gram negative bacteria.[30–32] ABPA diagnosis consisted of clinical deterioration, increased serum markers (total IgE>500 IU ml^–1^, elevated specific *Aspergillus* IgE and *Aspergillus* IgG), and new or recent abnormalities on chest radiographs.[33] All patients ever diagnosed with ABPA were included in the ABPA group since ABPA was considered a chronic condition independent of duration prior to the study, and with a continued risk of exacerbation.[33]

#### Pulmonary function tests

Spirometry and whole body plethysmography were performed according to ATS and ERS recommendations [34] using Jaeger Master Screen Pro (CareFusion, Hochberg, Germany). Reference equations from the Global Lung Initiative [35] and from Koopman et al. [36] were used to standardise spirometry outcomes, and static lung volume outcomes from plethysmography, respectively.

Nitrogen (N_2_) MBW measurements were performed using Exhalyzer D (Eco Medics AG, Duernten, Switzerland) in accordance with guidelines recommended in the recent ERS/ATS consensus statement.[37] Outcomes from MBW were calculated in Spiroware (v. 3.1.6 ext.; Eco Medics AG, Duernten, Switzerland) using standard software settings.

### Statistical analysis

Comparisons between groups of patients were performed using non-parametric Mann–Whitney tests (continuous variables) and Fisher’s exact test (frequencies). Spearman rank correlation and regression analyses were used to calculate the relation between pulmonary function test outcomes and CF-CT scores.

Statistical models and calculations were made with guidance from the statistical advisory service at the University of Copenhagen, using SAS Enterprise Guide version 5.1. (SAS Institute Inc. NC, USA). *P*-values <0.05 were considered significant.

### Ethics

This study was approved by the Danish National Committees on Biomedical Research Ethics, Capital Region of Copenhagen (Protocol no.: H-1-2010-042). The clinical database at the centre was approved by the Danish Data Protection Agency (2008-41-2682).

## Results

### Study population

A total of 78 children from the paediatric CF cohort counting *N *= 105 children were eligible based on age between 6 and 18 years, and 64 (82.1%,) were included in the study. Characteristics and main results of the study are outlined in Table 1. No significant differences were found between the included and the 14 non-included children (see Appendix (Figure A1) for details). Among the non-included children 12 simply declined (no explanations) to participate within the duration of the inclusion period, one refused due to fear of ionizing radiation, and in one the SCCCT seemed non-feasible during the training session.

**Table 1.** Demographics, CF-CT scores and pulmonary function test results.

Demographics	N	Median	Minimum	Maximum
Age (years)	64	12.7	6.4	18.1
BMI z-score	64	−0.2	−2.2	1.5
**CF-CT scores**				
CF-CT total score (%)	64	9.3	0.4	46.8
Bronchiectasis (%)	64	5.6	0.0	54.5
Airway wall thickening (%)	64	7.9	0.0	44.4
Mucus plugging (%)	64	1.4	0.0	52.8
Parenchyma (%)	64	0.9	0.0	11.1
Gas trapping (%)	64	40.7	3.7	100.0
**Pulmonary function tests**				
LCI	62	9.3	6.2	16.0
FEV_1_ % predicted	64	96.1	45.1	118.5
FVC % predicted	64	97.8	53.8	128.2
FEF_25-75_ % predicted	64	88.0	20.5	150.6
RV/TLC z-score	55	0.4	−1.9	3.2

**Table 2.** Correlation coefficients and regression values between outcomes of pulmonary function tests and CF-CT total scores.

Pulmonary function tests outcomes	r	R^2^
FEF_25-75_ %-pred.	−0.441*	0.192*
FEV_1_ %-pred.	−0.353*	0.242**
FVC %-pred.	−0.208	0.128*
LCI	0.739**	0.536**
RV/TLC z-score	0.607**	0.314**

FEF_25-75%:_ Forced expiratory flow within 25 and 75% of FVC. FEV_1_: forced expiratory volume in 1 sec. FVC: forced vital capacity. LCI: lung clearance index. RV: residual volume. TLC: total lung capacity. *p < 0.05, **p < 0.0001.

### CF-CT: Feasibility and scores

The feasibility of imaging using spirometry control was excellent (98.4%); only one examination showed motion artefacts due to inspiration during imaging manoeuvres (1.6%).

**Figure 1.**
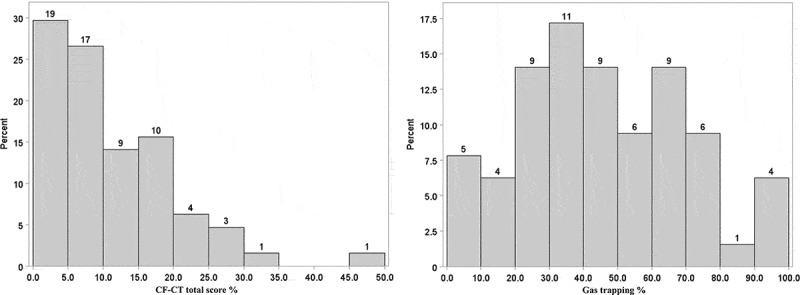
Frequency distribution of CF children according to: (a) CF-CT total score and (b) gas trapping score. Percentage (Y-axis) of the given score (X-axis) is shown as bars and absolute numbers on top of each bar.

The CF-CT scores, CF-CT total score and scores according to each domain, in the total group of 64 children are outlined in Table 1 and the frequency (%) distribution of the CF-CT total score is displayed in Figure 1(a). A large proportion of subjects exhibited marked gas trapping (Figure 1(b)), whereas the proportions showing changes in the other domains were very low (not shown).

### Microbiology: impact of infection status

**Figure 2.**
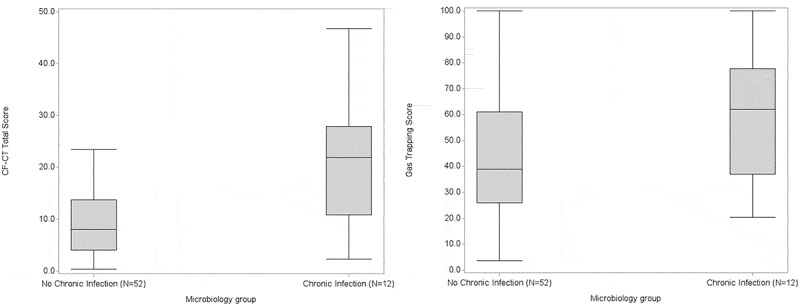
CF-CT total scores in % of maximal theoretical score and percentage of gas trapping according to infection status. (a) CF-CT total score, Mann–Whitney: *p *= 0.0028. (b) Gas trapping score, Mann–Whitney *p *= 0.0702. Data are presented as box and whiskers plots representing the 25th and 75th centiles and the range, respectively.

**Figure A2.**
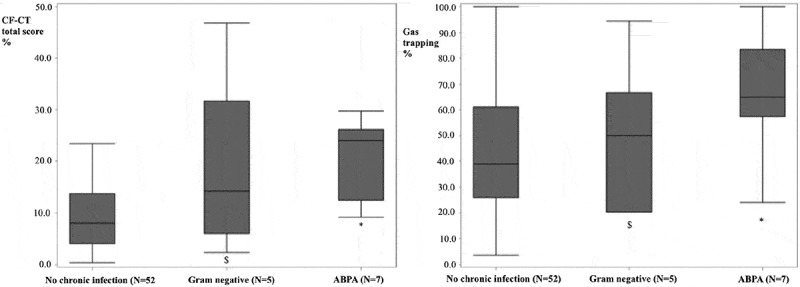
CF-CT total scores and percentage of gas trapping grouped according to infection status. (a) CF-CT total score, (b) gas trapping score. ^$^Non-significant, *p<0.01, compared to the non-infected group (Mann–Whitney test). Data are presented as box and whiskers plots representing the 25th and 75th centiles and the range, respectively.

Significantly higher median CF-CT total scores (21.9%) were found in patients with chronic infections (*N *= 12) compared with 8.0% in patients with no chronic lung infection, though with large variations in the scores (Figure 2(a)). The median CF-CT total score in the small groups with chronic Gram-negative infections (*N *= 5) and ABPA (*N *= 7) were 14.2% (NS) and 24.0% (*p *< 0.01), respectively (Appendix (Figure A1)). Marked gas trapping was shown in all groups irrespective of infection, thus exhibiting a median of 38.9% gas trapping in patients without infection and 62.0% (*p *< 0.01) with chronic infection, again showing large variation within groups (Figure 2(b) and Appendix (Figure A2)).

### Pulmonary function tests

**Figure 3.**
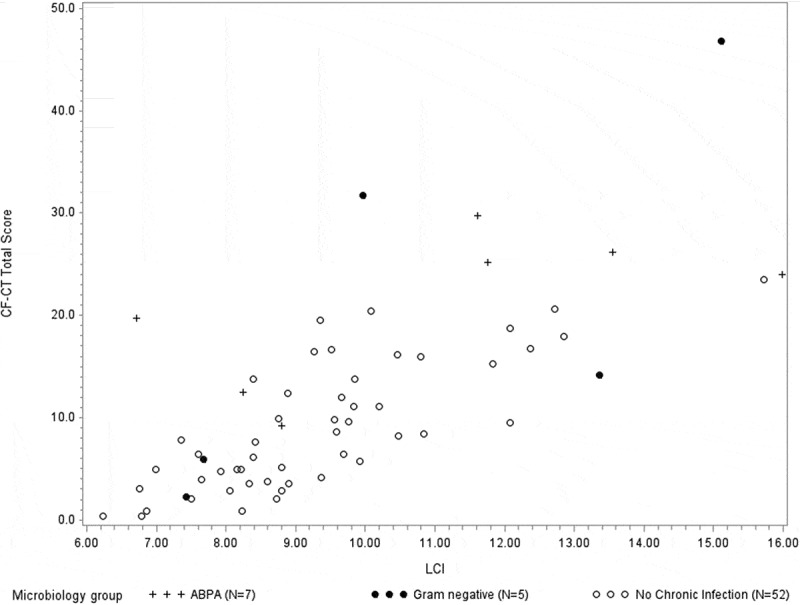
CF-CT total score (%) and the relationship to LCI (r = 0.739, *p *< 0.0001) in all *N *= 64 participants.

**Figure 4.**
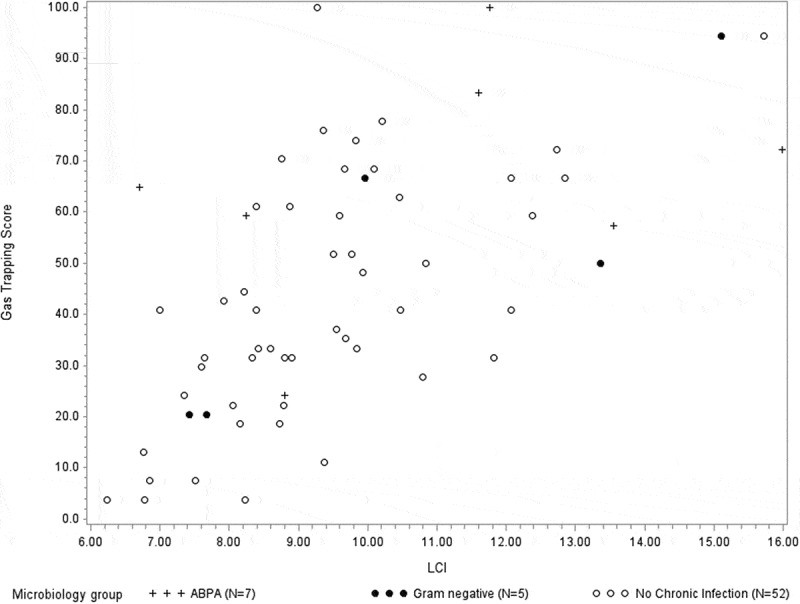
Gas trapping score (%) and the relationship to LCI (r = 0.633, *p *< 0.0001) in all *N *= 64 participants. Markers designate: no chronic infection, chronic Gram negative infection or ABPA as shown in legends.

The outcome of pulmonary function tests with the closest correlation to CF-CT total score and gas trapping was the LCI derived from MBW (Figures 3 and 4), and LCI was also the outcome with the highest regression value using linear regression, thus describing the highest amount of the variation in CF-CT total score (Table 2).

## Discussion

We investigated the structural lung changes in CF children with mild clinical disease using SCCCT refined by a computer-animated biofeedback system invented to further standardise lung volumes during CT examinations and to increase feasibility. The CF-CT total scores were generally low in this closely monitored CF cohort, whilst gas trapping was prominent in the majority of patients. Gas trapping has previously also been reported as a common abnormality in very young children with CF using volume controlled CT scans,[38] as well as in studies without breath hold control.[4] We have previously demonstrated the superiority of SCCCT to detect gas trapping in comparable cohorts of children examined without definite breath hold control displaying no or only very mild gas trapping in 19% of cases, compared to only 4.5% of cases in the cohort where volume control was used during CT imaging.[9] Accurate identification of structural lung changes is of great importance to the usefulness of CT examinations, especially for longitudinal studies. Controlling lung volume during imaging seems to be a tool that improves this accuracy.[8,39]

LCI was the pulmonary function outcome with the closest correlation to CF-CT total scores, which is in agreement with reports from other groups claiming LCI as being highly reliable in predicting CF structural lung damage.[4,20,22,23] Furthermore, we are reporting similar correlations between other pulmonary function outcomes and CF-CT scores in consistency with previous reports.[4,20,22,23]

The importance of CT as a supplementary tool in monitoring and management of children with CF is supported by the findings of this study since LCI only describes approximately half of the variation in the CF-CT total score. Thus valuable information would have been undiscovered if only LCI was used to predict the extent of structural lung changes.

An important strength of the study was the use of a computer-animated biofeedback to standardise breath hold. This method seems feasible in all age groups, and provides a high degree of consistency in the execution of all SCCCT examinations, which, in addition, were performed by a single person (TK). Good inter-observer agreement with an experienced observer regarding the CF-CT scoring system was confirmed on training CT scans from the Erasmus Medical Centre in Rotterdam, the Netherlands, prior to the scoring in this study, which has previously been proposed as an improvement in the utility of scoring systems.[40] Thus, the weakness of having only one observer was addressed, although assessment of intra-observer variability was not performed. Furthermore, the observer was blinded to the clinical condition and data during scoring.

The cross sectional design combined with retrospectively collected clinical data might have weakened the study, and longitudinal studies prospectively collecting such data are needed to investigate the associations between various infections, e.g. pulmonary aspergillosis and structural lung changes. With regard to the risk of selection bias, the analysis between the included and the excluded patients did in no way show any statically significant differences. The CF-CT scoring system might not have been the optimal system to quantify the extent of structural changes in this group of children with mild disease, where the majority received low scores. Other scoring systems developed explicitly for early disease, such as the PRAGMA system,[41] might prove to be superior to the CF-CT scoring system in such cases.

A refined SCCCT using computer-animated biofeedback was applied in this study to standardise breath hold during CT imaging. Significant prominence of gas trapping was shown in CF children with mild disease but the clinical impact of this is not yet known and further longitudinal studies using SCCCT or other methods of breath hold control are needed for verification.

Despite the low number of chronically infected children we did explore the impact of chronic Gram-negative lung infections and ABPA on the presence of structural lung changes and found an overall significant impact attributable to ABPA. Future, larger studies should investigate manifestations of *Aspergillus fumigatus* infection with or without allergy since the impact of this is less substantiated.[17–19]

In conclusion the most prominent structural lung change was gas trapping, while CF-CT total scores were generally low. Chronic lung infections, specifically in the form of ABPA, were associated with increased lung changes. The CT examinations cannot be fully replaced by any lung function measurements such as LCI, or other clinical markers in the detection of pulmonary damage in CF. Further investigation into impact of infections with different microorganisms on extent and progression of structural CF lung disease is needed.

## Supplementary Material

Supplementary Material

## Appendix

### Computed tomography

All scans were performed by volumetric spiral CT imaging on a Toshiba Aquillion 64 CT scanner (Toshiba Corporation, Tokyo, Japan; 100 kVp, mAs-modulation; SD = 19 in inspiratory and SD = 27 in expiratory sequences, rotation 0.4 s). The average effective dose for both inspiratory and expiratory CT was 1.56 mSv (range 0.76–4.05) and calculated using age-specific conversion factors (E1).

### CF-CT scoring

Good inter-observer agreement with a reference observer was secured prior to scoring the study cohort, using training CTs that were scored by an experienced reference scorer.

Inter-observer agreement on training CF-CT scores was calculated using intra-class correlation coefficients for calculated total and subdomain scores, supplemented by weighted kappa statistics on individual observations for subdomains (coefficients ≥ 0.8 indicate excellent agreement, 0.6–0.8 good agreement, 0.4–0.6 moderate agreement, 0.2–0.4 fair agreement, and <0.2 poor agreement), and Bland–Altman plots to display signs of systematic bias in subdomains.

Inter-observer agreement on training CT scoring using intra-class correlation coefficients ranged from good (AT and airway wall thickening: 0.77) to excellent (CF-CT total score: 0.88).

The weighted kappa statistics on every individual observation ranged from moderate (0.43 (AWT), 0.48 (parenchyma), 0.53 (AT)) to good (0.71 (BE), 0.61 (mucus plugging), 0.67 (all scores combined)). Bland–Altman plots showed no sign of systematic bias in any of the sub-scores (Fig. E1).

### Results

The included cohort was compared to the non-included subjects and exhibited no significant differences, though no patients in the non-included group had ABPA or chronic *P. aeruginosa* infection (Table E1).

**Table A1.** Comparison between study cohort and excluded subjects.

	Study cohort	*n*=64	Non-included	*n*=14	Test
Subjects	Numbers	%	Numbers	%	*p*
Males	34	53	7	50	0.2
Homozygote ∆508	33	52	8	57	0.2
Chronic *P. aeruginosa*	5	6	0	0	0.4
*ABPA*	7	13	0	0	0.2
FEV_1_<80%	13	20	3	21	0.3
FVC<80%	5	8	2	14	0.3
					
*Other characteristics (means)*	Study cohort		Non-included		*p*
Age (years)	12.3		13.3		0.3
BMI-z scores	-0.1		-0.5		0.1
*P. aeruginosa* IgG precipitins	2.0		3.0		0.5
*A. fumigatus* IgG (EU)	48.7		55.3		0.8
S. aureus %positive cultures/year	33.9		34.0		1.0

ABPA: allergic bronchopulmonary aspergillosis. BMI: body mass index. EU: Elisa units.

## Reference

1. E1. Deak PD, Smal Y, Kalender WA. Multisection CT protocols: sex- and age-specific conversion factors used to determine effective dose from dose-length product. Radiology. 2010; 257:158-66. doi: 10.1148/radiol.10100047.
