# Supplementary material for: Association between spirometry controlled chest CT scores using computer-animated biofeedback and clinical markers of lung disease in children with cystic fibrosis
Source: Eur Clin Respir J. 2017 May 2;4(1):1318027. doi: 10.1080/20018525.2017.1318027 (PMC5475300; doi:10.1080/20018525.2017.1318027)
Supplement: Supplementary Material [file zecr_a_1318027_sm2232.pdf]

Computed tomography:

All scans were performed by volumetric spiral CT imaging on a Toshiba Aquillion 64 CT scanner (Toshiba Corporation, Tokyo, Japan; 100 kVp, mAs-modulation; SD = 19 in inspiratory and SD = 27 in expiratory sequences, rotation 0.4 s). The average effective dose for both inspiratory and expiratory CT was 1.56 mSv (range 0.76– 4.05) and calculated using age-specific conversion factors (E1).

CF-CT scoring:

Good inter-observer agreement with a reference observer was secured prior to scoring the study cohort, using training CT's that were scored by an experienced reference scorer.

Inter-observer agreement on training CF-CT scores was calculated using intra-class correlation coefficients for calculated total and subdomain scores, supplemented by weighted kappa statistics on individual observations for subdomains (coefficients ≥ 0.8 indicate excellent agreement, 0.6-0.8 good agreement, 0.4 – 0.6 moderate agreement, 0.2 – 0.4 fair agreement, and <0.2 poor agreement), and Bland-Altman plots to display signs of systematic bias in subdomains.

Inter-observer agreement on training CT scoring using intra-class correlation coefficients ranged from good (AT and airway wall thickening: 0.77) to excellent (CF-CT total score: 0.88).

The weighted kappa statistics on every individual observation ranged from moderate (0.43 (AWT), 0.48 (parenchyma), 0.53 (AT)) to good (0.71 (BE), 0.61 (mucus plugging), 0.67 (all scores combined)). Bland-Altman plots showed no sign of systematic bias in any of the sub-scores (Fig. E1).

Fig. E1 Bland-Altman plots for total score and subdomains assessing systematic bias between scorers in the training CTs done prior to scoring the study cohort.

Total score κ=0.67	Bronchiectasis score κ=0.71	Airway wall score κ=0.43
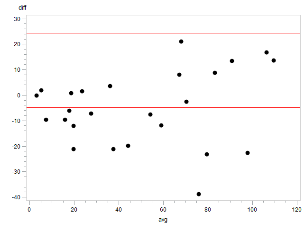	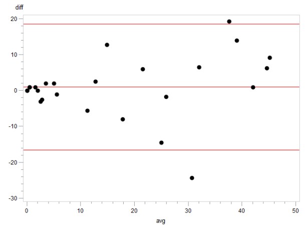	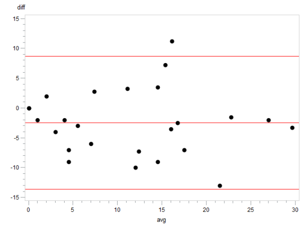
Mucus plugging score κ=0.61	Parenchymal change score	Air trapping score κ=0.53

	κ=0.48	
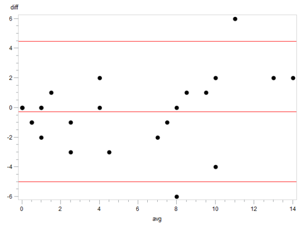	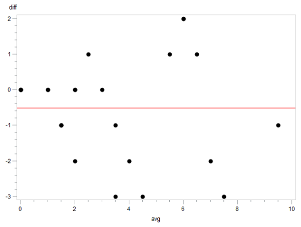	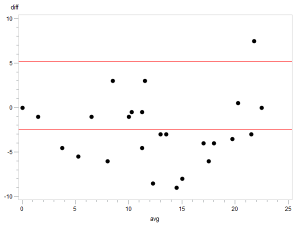

Results:

Comparisons of the study cohort with the non-included subjects:

The included cohort was compared to the non-included subjects and exhibited no significant differences, though no patients in the non-included group had ABPA or chronic *P. aeruginosa* infection (Table E1).

Table E1 Comparison between study cohort and excluded subjects.

**Subjects**	**Study cohort**	**n=64**	**Non-Included**	**n=14**	**Test**
	**Numbers**	**%**	**Numbers**	**%**	**p**
**Males**	34	53	7	50	0.2
**Homozygote del-508**	33	52	8	57	0.2
**Chronic *P. aeruginosa***	5	6	0	0	0.4
**Chronic *S. aureus***	21	33	6	43	0.2
**ABPA**	7	13	0	0	0.2
**FEV_1<80%**	13	20	3	21	0.3
**FVC<80%**	5	8	2	14	0.3
**Other outcomes (means)**	**Study cohort**		**Non-included**		**t-test p**
**Age**	12.3		13.3		0.3
**BMI-z scores**	-0.1		-0.5		0.1
***P. aeruginosa* IgG**	2.0		3.0		0.5
***Aspergillus* IgG**	48.7		55.3		0.8
***S. aureus* %**	33.9		34.0		1.0

ABPA: Allergic bronchopulmonary aspergillosis. BMI: Body mass index.

References

E1. Deak PD1, Smal Y, Kalender WA. Multisection CT protocols: sex- and age-specific conversion factors used to determine effective dose from dose-length product. Radiology. 2010; 257:158-66. doi: 10.1148/radiol.10100047.
